# An investigation of the association between focal damage and global network properties in cognitively impaired and cognitively preserved patients with multiple sclerosis

**DOI:** 10.3389/fnins.2023.1007580

**Published:** 2023-02-07

**Authors:** A. L. Wenger, Muhamed Barakovic, Sara Bosticardo, Sabine Schaedelin, Alessandro Daducci, Simona Schiavi, Matthias Weigel, Reza Rahmanzadeh, Po-Jui Lu, Alessandro Cagol, Ludwig Kappos, Jens Kuhle, Pasquale Calabrese, Cristina Granziera

**Affiliations:** ^1^Translational Imaging in Neurology (ThINk) Basel, Department of Biomedical Engineering, University Hospital Basel, University of Basel, Basel, Switzerland; ^2^Interdisciplinary Platform, Psychiatry, and Psychology, Division of Molecular and Cognitive Neuroscience, Neuropsychology, and Behavioral Neurology Unit, University of Basel, Basel, Switzerland; ^3^Neurologic Clinic and Policlinic, MS Center and Research Center for Clinical Neuroimmunology and Neuroscience Basel (RC2NB), University Hospital Basel, University of Basel, Basel, Switzerland; ^4^Department of Computer Science, University of Verona, Verona, Italy; ^5^Clinical Trial Unit, Department of Clinical Research, University Hospital Basel, University of Basel, Basel, Switzerland; ^6^Division of Radiological Physics, Department of Radiology, University Hospital Basel, Basel, Switzerland

**Keywords:** multiple sclerosis (MS), connectomics, structural connectivity, neuropsychological test, information processing speed

## Abstract

**Introduction:**

The presence of focal cortical and white matter damage in patients with multiple sclerosis (pwMS) might lead to specific alterations in brain networks that are associated with cognitive impairment. We applied microstructure-weighted connectomes to investigate (i) the relationship between global network metrics and information processing speed in pwMS, and (ii) whether the disruption provoked by focal lesions on global network metrics is associated to patients’ information processing speed.

**Materials and methods:**

Sixty-eight pwMS and 92 healthy controls (HC) underwent neuropsychological examination and 3T brain MRI including multishell diffusion (dMRI), 3D FLAIR, and MP2RAGE. Whole-brain deterministic tractography and connectometry were performed on dMRI. Connectomes were obtained using the Spherical Mean Technique and were weighted for the intracellular fraction. We identified white matter lesions and cortical lesions on 3D FLAIR and MP2RAGE images, respectively. PwMS were subdivided into cognitively preserved (CPMS) and cognitively impaired (CIMS) using the Symbol Digit Modalities Test (SDMT) z-score at cut-off value of −1.5 standard deviations. Statistical analyses were performed using robust linear models with age, gender, and years of education as covariates, followed by correction for multiple testing.

**Results:**

Out of 68 pwMS, 18 were CIMS and 50 were CPMS. We found significant changes in all global network metrics in pwMS vs HC (*p* < 0.05), except for modularity. All global network metrics were positively correlated with SDMT, except for modularity which showed an inverse correlation. Cortical, leukocortical, and periventricular lesion volumes significantly influenced the relationship between (i) network density and information processing speed and (ii) modularity and information processing speed in pwMS. Interestingly, this was not the case, when an exploratory analysis was performed in the subgroup of CIMS patients.

**Discussion:**

Our study showed that cortical (especially leukocortical) and periventricular lesions affect the relationship between global network metrics and information processing speed in pwMS. Our data also suggest that in CIMS patients increased focal cortical and periventricular damage does not linearly affect the relationship between network properties and SDMT, suggesting that other mechanisms (e.g. disruption of local networks, loss of compensatory processes) might be responsible for the development of processing speed deficits.

## Highlights

– Microstructural connectomics showed a decrease in global efficiency, clustering coefficient, network density, and mean strength in pwMS compared to healthy subjects.
– In pwMS, the combination of lesion type [periventricular, cortical (especially leukocortical)], and global graph metrics (density/modularity) influenced SDMT performance.
– In CIMS patients increased focal cortical and periventricular damage does not influence the relationship between SDMT and global graph metrics.

## 1. Introduction

Multiple Sclerosis (MS) is a chronic, autoimmune, neurodegenerative, and demyelinating disease of the central nervous system causing sensory and motor deficits, fatigue and cognitive impairment (Sparaco et al., 2021).

Impairment in cognitive functions occurs in 40–65% patients with MS (pwMS) (Jongen et al., 2012) and can be seen in all subtypes of MS. PwMS may suffer from cognitive deficits encompassing executive function, complex attention, episodic memory and information processing speed (Filippi et al., 2010). Information processing speed is a measure of the efficiency of cognitive functions (Sweet, 2011) and its impairment has been reported in approximately 40–70% of pwMS (Filippi et al., 2010). Besides, it is one of the first cognitive deficits to be observed in cognitively impaired MS patients (CIMS) and correlates strongly with working memory performance (Giorgio and De Stefano, 2010; DeLuca et al., 2015). The Symbol Digit Modalities Test (SDMT) is considered the gold standard to assess processing speed and working-memory related cognitive performance in pwMS and to discriminate between HC and pwMS (Langdon et al., 2012; Benedict et al., 2017).

Diffusion magnetic resonance imaging (dMRI) provides images that can be exploited to reconstruct the direction of fiber bundles in the brain (tractography) (Sporns et al., 2005) and the organization of neuronal networks (connectomics) (Sporns et al., 2005; Kocevar et al., 2016; Welton et al., 2020). Previous work using connectomics in pwMS showed that brain network dysfunction contributes to cognitive impairment (Dineen et al., 2009; Schoonheim et al., 2015).

Other studies comparing structural network changes and information processing speed in pwMS and HC have shown that networks in pwMS patients are less efficient (Schoonheim et al., 2015; Shu et al., 2016; Charalambous et al., 2019; Bosticardo et al., 2021), and that pwMS have an increase in network segregation and inversely a decrease in network integration (Van Schependom et al., 2014; Charalambous et al., 2019; Welton et al., 2020). A recent study by Welton and colleagues also reported that pwMS with low SDMT score had reduced “small-worldness” (i.e., less efficient information segregation and integration), lower global efficiency and longer average path lengths compared to healthy controls (Welton et al., 2020). All these studies were performed using connectomes weighted by the number of streamlines (NOS) connecting couples of brain areas; yet, we have recently shown that microstructure-weighted connectomes provide more sensitive measure of MS pathology compared to NOS- weighted connectomes (Bosticardo et al., 2021).

Focal cortical pathology is correlated with the development of cognitive impairment in pwMS. In fact, cortical lesion volume correlates with information processing speed as measured by the SDMT (Papadopoulou et al., 2013). Further, cortical subpial pathology, as measured by a T_2_* increase at 7T MRI, appears to be strongly associated with cognitive function in pwMS in a way that is independent from white matter pathology and diffuse cortical damage, as quantified using cortical thickness (Forn et al., 2011; Louapre et al., 2016). In addition, alterations in T2* across the whole cortex relate to lower performance in SDMT (Forn et al., 2011). On the other hand, the number of juxtacortical lesions in pwMS correlates with a global scale of cognitive impairment (Lazeron et al., 2000), and those lesion types seem to be also associated with specific deficits in information processing speed (Forslin et al., 2018).

A role of white matter (WM) lesions in cognitive impairment has also been established in previous works (Papadopoulou et al., 2013); yet to date, the relationship between the location of WM lesions, global brain network properties alterations and cognitive impairment has not been investigated.

In this work, we used microstructure-weighted connectomics to explore the hypothesis that MS lesion types affect the relationship between global brain network metrics and cognitive performance. Our specific aims were to (i) assess differences in global network metrics (modularity, density, efficiency, mean strength, and clustering coefficient) obtained with microstructure-weighted connectomics between pwMS and HC; (ii) to investigate the relationship between those global network metrics and cognition measured with SDMT in pwMS; and finally (iii) to assess if this relationship is influenced by either volume or number of subtypes of cortical or white matter lesions.

## 2. Materials and methods

### 2.1. Study participants

We enrolled pwMS recruited between January 2019 and June 2021 within the INsIDER study at the University Hospital of Basel (Basel, Switzerland). Main inclusion criteria for this study were: age between 18 and 75 years, MS diagnosis (RRMS, PPMS, and SPMS) fulfilling revised 2017 McDonald criteria (Thompson et al., 2018), absence of severe psychiatric condition or neurological disease other than MS. Progressive MS patients were non-active (i.e., no relapses and no MRI activity) and relapsing-remitting MS patients were active but the MRI was performed with a 3-month delay from the last relapse.

Exclusion criteria were pregnancy, contraindication (e.g., claustrophobia, pacemaker) to magnetic resonance imaging (MRI) and inability to give informed consent. All subjects, healthy controls and pwMS, underwent neuropsychological examination and advanced MRI at 3T. Participants were excluded if SDMT, fatigue questions, demographic variables were missing or if data did not pass strict quality check. The Ethic Review Committee of the University of Basel approved the INsIDER study (registration number: NCT05177523) and all participants signed informed consent. Only few patients were recruited after the beginning of the COVID-19 pandemic in Europe in March 2020 with no apparent changes in the neuropsychological testing.

### 2.2. Clinical assessment and patients’ stratification

Patients with multiple sclerosis (PwMS) and HC underwent a neurological and neuropsychological assessment including the oral version of the Symbol Digit Modalities Test (SDMT) (Smith, 1968) and MUSIC test (Kalbe et al., 2013). Within the MUSIC test three questions on fatigue; subdivided into cognitive and physical fatigue and fatigue impacting work and social life were asked. We examined the HADS (Hospital Anxiety and Depression Scale) questionnaire that were collected at the time of the cognitive examination in our cohort of pwMS (Zigmond and Snaith, 1983). We then calculated whether symptoms of depression, anxiety and fatigue differed between cognitively preserved and cognitively impaired MS patients using the Mann-Whitney *U*-Test. SDMT raw scores (Kurtzke, 1983) were used for the statistical analysis. Clinical disability in pwMS was assessed with the Expanded Disability Status Scale (EDSS) (Kurtzke, 1983). PwMS were divided into cognitively impaired (CIMS) and cognitively preserved patients (CPMS) using the SDMT z-score at −1.5 standard deviations (Lorefice et al., 2021). PwMS and HCs were asked the number of years of educations in the neuropsychological assessment.

### 2.3. MRI data acquisition

All subjects underwent brain MRI on a 3T system (Magnetom Prisma, Siemens Healthcare, Erlangen, Germany) with a 64-channel head and neck coil for RF reception. The acquisition protocol included (i) 3D FLuid Attenuated Inversion Recovery [FLAIR, repetition time (TR)/ echo time (TE)/ inversion time (TI) = 5,000/386/1,800 ms] 1 mm^3^ isotropic spatial resolution; (ii) 3D Magnetization Prepared 2 Rapid Acquisition Gradient Echoes (MP2RAGE, TR/TI1/TI2 = 5,000/700/2,500 ms) 1 mm^3^ isotropic spatial resolution; (iii) multi-shell diffusion [TR/TE/impulse duration (d)/time between impulses (D) = 4,500/75.0/19.2/36.5 ms] 1.8 mm^3^ isotropic spatial resolution with *b*-values 700/1,000/2,000/3,000 s/mm^2^ and 6/20/45/66 diffusion directions, respectively, per shell, and 12 measurements at *b*-value 0 s/mm^2^ with both anterior to posterior as well as reversed phase encoding. Diffusion MRI images were pre-processed including denoising, motion correction, eddy currents and bias field correction as described in Bosticardo et al. (2021).

### 2.4. MS lesion identification and connectomics

White matter and cortical MS lesions were segmented with an automatic deep learning-based method (La Rosa et al., 2020), followed by manual correction by two expert raters. Lesion-filled MP2RAGE were processed with FreeSurfer^1^ and the standard Desikan-Killiany atlas was used for the automatic segmentation that provides cortical and subcortical parcellation of 85 regions of interest (ROIs). We identified as juxtacortical lesions, areas of T2-hyperintensities that are abutting the cortex without intervening normal-appearing white matter. To facilitate the identification of these lesions, we automatically segmented an area of 3 mm from the cortex, which covers the entire thickness of the U-fibers. Lesions in this area were then manually identified as juxtacortical or not.

We considered periventricular lesions those that were located within a 3-mm boundary from the ventricles. If confluent, periventricular lesions were manually split by an experienced neuroradiologist and neurologist (when possible) or counted as one (when not possible to split them). Intracortical and leukocortical lesions were identified manually on MP2RAGE images using the lesion map obtained as detailed above. With currently available techniques it is challenging to detect intracortical lesions programmatically. They were therefore assessed through individual inspection by a trained neuroradiologist and neurologist. Each leukocortical lesion was split into its white matter and gray matter components on MP2RAGE images by two trained experts. MP2RAGE images provide in fact a sharp contrast between cortical gray and the underlying white matter, which is facilitating this task. We performed this anatomical distinction to better understand whether the pathology affecting the inner cortical layers or the one impacting the function of the underlying U-fibers best correlates with processing speed as measured with SDMT.

Connectomes were reconstructed using MRtrix3 (Tournier et al., 2019) and were weighted by a microstructural map that was derived from the Spherical Mean Technique (SMT), which is a technique used for microscopic diffusion anisotropy imaging to map microstructural features that are not confounded by orientation dispersion or fiber crossing (Kaden et al., 2016; Bosticardo et al., 2021). For the estimation of the Neurite Volume Fraction map (INTRA) (Bosticardo et al., 2021) derived from SMT, an open-source code was used.^2^

### 2.5. Graph metrics estimation

Using Brain Connectivity Toolbox,^3^ we extracted the following global network metrics in brain connectomes weighted by INTRA: (i) *Modularity*, which measures the capability of a network to be divided into modules and reflects the network segregation; (ii) *Density*, which is a measurement of the ratio between the actual and possible connections (iii) *Clustering coefficient*, which describes the tendency of two neighboring nodes to be connected to a common node; (iv) *Mean strength*, which measures the average sum of edge weights that are connected to a node; and (v) *Efficiency*, which measures the capability of the exchange of information across the whole network (Rubinov and Sporns, 2010; Zalesky et al., 2010; Sporns, 2013; Kocevar et al., 2016; Bosticardo et al., 2021).

### 2.6. Statistical analysis

We assessed the following H0-hypotheses:

• H0-1: There is no difference between individual global network metrics in pwMS and HC.
• H0-2: There is no correlation between global network metrics and information processing speed in pwMS and HC.
• H0-3: The combination of global network metrics, cortical (CL) or white matter (WM) lesion number or volume is not related to information processing speed in pwMS.

All statistical analyses were done using R Studio (R Core Team, 2017) with the help of a senior statistician (SS).

To evaluate whether variables were normally distributed, we used the Shapiro-Wilk test. Demographics and clinical variables were assessed with *t*-tests if variables were normally distributed or with the Mann-Whitney *U*-test (e.g., EDSS, fatigue/depression/anxiety scores or lesion number and volume) if variables did not follow normal distribution.

With a principal component analysis (PCA), we first attempted at reducing the dimensionality of the variables that we planned to compare between pwMS and HCs.

Then, H0-1 was confuted using linear robust models controlling for eventual outliers with age, gender, patient’s years of education and network density as covariates. Density was considered as covariate, since recent studies have shown that the network density strongly differs among pwMS and HC (Schiavi et al., 2020a,b; Bosticardo et al., 2021). We reported adjusted *p*-values with Holm correction for multiple testing in Table 2.

**TABLE 1:** Descriptive analysis of demographics and clinical variables in patients with multiple sclerosis (pwMS) and healthy controls (HC).

Variables	Healthy controls (HC)	Multiple sclerosis (pwMS)	*P*-values
N/Gender	92 (48 females)	50 (30 females)	*p* < 0.01
Age	36.2 (12.9)	46.8 (14.4)	*p* < 0.01
Education (years)	17.7 (3.63)	16.5 (4.60)	*p* < 0.05
EDSS		3.11 (1.88)	
Disease duration in years		11.3 (11.8)	
SDMT score (*)	64.1 (13.0)	56.2 (15.4)	*p* < 0.01
SDMT *z*-score	−0.0978 (1.04)	−0.529 (1.28)	*p* < 0.01

EDSS, Expanded Disability Status Scale; SDMT, Symbol Digit Modalities Test. EDSS was measured with Expanded Disability Status Scale (Kurtzke, 1983). SDMT *z*-Scores were calculated with standardized tables from Smith (1968) controlling for age and education. For age, years of education, SDMT score, SDMT *z*-score and disease duration mean, and standard deviations were calculated. We have calculated *p*-values with *t*-tests if variables followed normal distribution (*) and with Mann-Whitney *U*-test if they did not follow normal distribution.

**TABLE 2:** Specific comparisons between healthy controls (HC) and patients with multiple sclerosis (pwMS) and each global network.

Variables	Contrasts	Estimate	Standard error	*t*-ratio	Adjusted *P*-value	Multiple R^2^ and adjusted R^2^
Density	HC-MS	0.0215	0.00612	3.518	0.0006^a^	0.194 (0.1732)
Efficiency	HC-MS	0.0129	0.00413	3.130	0.0021^a^	0.5316 (0.5164)
Clustering coefficient	HC-MS	0.0121	0.00399	3.030	0.0029^a^	0.5521 (0.5376)
Mean strength	HC-MS	0.91	0.288	3.164	0.0019^a^	0.7513 (0.7433)
Modularity	HC-MS	−0.0021	0.00169	−1.247	0.2144	0.7648 (0.7571)

For each global graph metric, comparisons between the two groups, HC and MS, were calculated. Global graph metrics differed between healthy controls MS patients. Except for modularity, these comparisons were highly significant. Adjusted *p*-values using holm correction are reported. HC, healthy controls; MS, multiple sclerosis; SE, standard error.

^a^Significantly different at *p* <0.01.

To confute H0-2 we calculated Spearman correlations between each global graph metric and information processing speed in pwMS and HC.

To assess H0-3 we tested whether lesion types (CL or WM) and lesion characteristics (lesion volume or number), and global graph metrics were associated with information processing speed in pwMS using linear robust models followed by Bonferroni correction.

In an exploratory way, we then assessed which combination of variables is best related to SDMT in a subgroup of patients, namely CPMS and CIMS. To achieve this goal, we performed linear robust models for each condition, where SDMT score was considered as dependent variable whereas lesion type (juxtacortical, periventricular, leukocortical GM and leukocortical WM, and intracortical) and lesion volume or number together with global graph metrics were the independent variables. Gender, education, and age were applied as covariates.

For all hypotheses, a two-sided *p*-value of ≤ 0.05 and a confidence interval of 0.95 was considered as statistically significant.

## 3. Results

### 3.1. Study population

**FIGURE 1:**
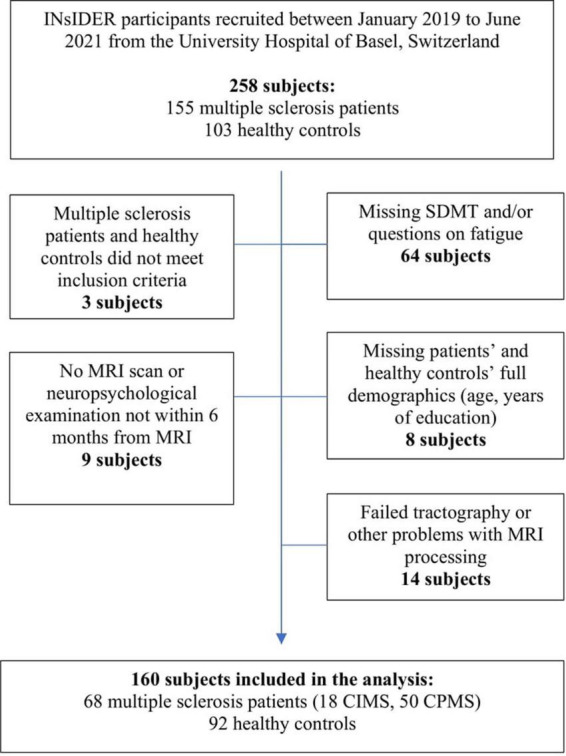
Consort flow diagram of participants who met inclusion and exclusion criteria. In total 160 subjects were included in the analysis, 68 were patients with multiple sclerosis (pwMS), and 92 healthy controls (HC).

Out of 155 INsIDER pwMS and 103 INsIDER HC, 68 pwMS and 92 HC were eligible for participation. PwMS and HC underwent neuropsychological examination and MRI usually within 60 days. The results of the oral SDMT z-scores were used to determine CPMS and CIMS categories, resulting in 50 CPMS (30 females) and 18 CIMS (12 females) patients. Figure 1 displays the consort flow diagram of participants who met inclusion criteria for the study population.

### 3.2. Baseline characteristics and results of neuropsychological testing

Demographic variables of HC and pwMS are depicted in Table 1. HC were younger and had more years of education than MS patients. Compared to pwMS, HC had on average a higher SDMT score. CIMS and CPMS had lower SDMT z-scores, and raw-scores (Supplementary Table 1) compared to HC. CIMS had a longer disease duration compared to CPMS and EDSS differed significantly between CPMS and CIMS patients (*p* < 0.001, Supplementary Table 1).

Multiple sclerosis (MS) subtypes in CPMS and CIMS are shown in Supplementary Table 1. The majority of the CPMS subgroup patients suffered from relapsing-remitting multiple sclerosis (RRMS) (80%, 40 patients), whereas clinical MS subtypes were equally represented in the CIMS subgroup (Supplementary Table 2).

Total Fatigue scores, as measured within the MUSIC questionnaire differed significantly between the two patient groups (*p* < 0.05). However, the subscale of cognitive fatigue did not show significant differences between CPMS and CIMS patients (*p* = 0.1472). Interestingly, motor fatigue differed significantly between groups with more difficulties in CIMS in comparison to CPMS patients (*p* < 0.05). Symptoms of depression (*p* = 0.176) and anxiety (*p* = 0.926) did not differ between CPMS and CIMS.

At the time of MRI, most patients were treated with Ocrelizumab (44,1%), followed by Dimethyl fumarate (14,7%), Rituximab (10,3%), Fingolimod (8,82%), Teriflunomide (4,41%), Natalizumab (2,94%), Interferon-beta (2,94% and 1,47%), and Sativex Spray (1,47%). Six pwMS were not treated with medications at time of MRI (8,82%).

### 3.3. Group differences in network organization

**FIGURE 2:**
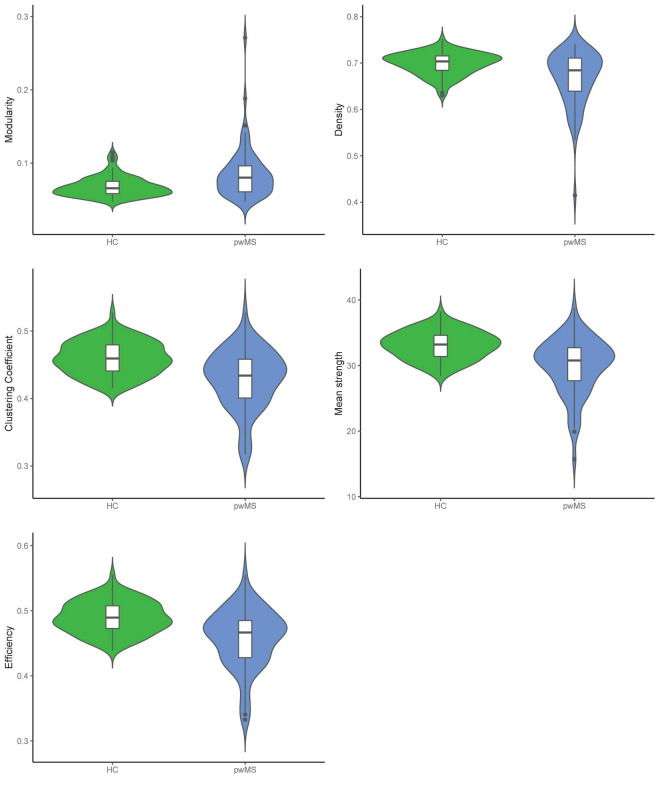
Violin plots of the five global network metrics for healthy controls (HC) and patients with multiple sclerosis (pwMS). The figure shows violin plots between the two groups and the five global network metrics. Bars in the middle represent median values. The whiskers show 95% confidence interval, and the shape of each violin plot displays the frequencies of values, here: number of participants with same global network value.

Differences in global graph metrics between pwMS and HC are reported in Table 2 and can be visually seen in Figure 2. Density, efficiency, clustering coefficient, and mean strength were lower in pwMS compared to HC. All global graph metrics were significantly different between pwMS and HC, except for modularity. Density, efficiency, clustering coefficient, and mean strength were lower in CIMS/CPMS patients compared to HC but did not differ between the two patients’ subgroups except for mean strength (Supplementary Table 3).

### 3.4. Correlation between SDMT and global network metrics

**FIGURE 3:**
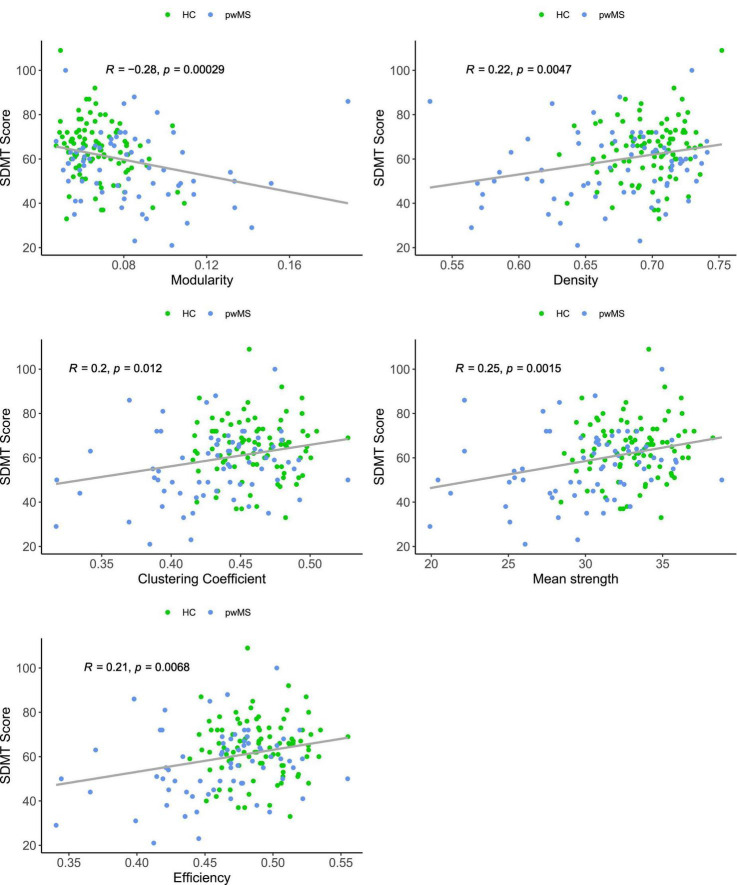
Scatter plots between symbol digit modalities test (SDMT) score and global network metrics, color-coded for the two groups [patients with multiple sclerosis (pwMS) and healthy controls (HC)]. Spearman’s correlation coefficient suggests an association between each global network metric and information processing speed in all participants. Correlations are positive for density, clustering coefficient, mean strength and efficiency, and negative for modularity.

Across all participants (pwMS and HC), all five global network metrics significantly correlated with SDMT score. Correlations with SDMT were positive for density (*r* = 0.22, *p* = 0.0047), clustering coefficient (*r* = 0.2, *p* = 0.012), mean strength (*r* = 0.25, *p* = 0.0015) and efficiency (*r* = 0.21, *p* = 0.0068). Modularity and SDMT scores correlated negatively (*r* = −0.28, *p* = 0.00029). Depending on each group, correlations varied between SDMT and each global graph metrics (Figure 3). We found the strongest association between clustering coefficient (*r* = 0.268), efficiency (*r* = 0.302) and mean strength (*r* = 0.268) and SDMT in CIMS patients compared to the overall patient group and HCs.

### 3.5. MS lesion load in CPMS and CIMS

White matter (WM) lesions were more frequent, and their total volume was larger in CIMS compared to CPMS patients (Table 3). Also, the number of juxtacortical lesions as well as the number of periventricular and volume of periventricular lesions were higher in CIMS in comparison to CPMS patients (Table 3). A visualization of the range for different lesion types (number and volume) can be found in the Supplementary Figure 2.

**TABLE 3:** Analysis of lesion types in patients with multiple sclerosis (pwMS).

Variables	Cognitively preserved MS (CPMS)	Cognitively impaired MS (CIMS)	*P*-value	95% CI
White matter–volume	3.17 [6.64]	9.69 [7.13]	0.00038	4.0039–9.2149
White matter–number	39 [44]	54 [33.5]	0.03116	2.0000–36.9999
Juxtacortical–volume	1.11 [2.54]	3.42 [4.14]	0.00106	1.2039–3.28199
Juxtacortical–number	31.5 [40.2]	63 [51.2]	0.00255	12.000–47.999
Periventricular–volume	0.409 [1.05]	2.28 [2.23]	0.00101	0.4820–2.1489
Periventricular–number	12 [12.5]	19.5 [7]	0.00752	1.9999–12.9999
Cortical–volume	0.0335 [0.224]	0.272 [0.389]	0.003844	0.0200–0.2719
Cortical–number	1 [5.75]	8.5 [13.5]	0.001847	1.0000–9.9999
Intracortical–volume	0 [11.5]	17.5 [63.8]	0.05051	−9.354e-06–2.404e+01
Intracortical–number	0 [1]	1 [3.75]	0.05503	−5.259e-05–1.999
Leukocortical–volume WM	9.5 [106]	78 [200]	0.00849	5.999–97.000
Leukocortical–volume GM	10 [73.8]	117 [174]	0.00357	11.000–119.999
Leukocortical–number	1 [4]	7.5 [11]	0.001873	1.000–8.999

Unit of lesion volume: mm^3^, values are expressed as median and interquartile range. Leukocortical–volume WM, leukocortical–volume white matter; leukocortical–volume GM, leukocortical volume gray matter. *P*-values and 95% CI were calculated using Mann-Whitney *U*-test.

### 3.6. Principal component analysis of global graph metrics

Principal component analysis for the five global graph metrics revealed high collinearity for efficiency and clustering coefficient (*r* = 0.992), modularity and density (*r* = −0.805) and efficiency and mean strength (*r* = 0.943). In contrast, efficiency and density (*r* = 0.6019), efficiency and modularity (*r* = −0.500) and clustering coefficient and modularity (*r* = −0.530) correlated only moderately with each other (Supplementary Figure 1); Because of the challenges in interpreting PCA results, we have however chosen to not calculate one combined global graph metric score for all pwMS but to perform linear robust models to assess each graph metric independently, followed by correction for multiple testing (see Section “3.7. Influence of MS lesions on the association between global graph metrics and SDMT”).

### 3.7. Influence of MS lesions on the association between global graph metrics and SDMT

In pwMS the combination of lesion type (cortical or white matter) and lesion characteristics (number or volume) together with global graph metrics influenced SDMT.

We found an independent association between cortical lesion volume, density and SDMT in pwMS (adjusted *p*-value: 0.00038). Furthermore, modularity and cortical lesion volume influenced SDMT in pwMS (adjusted *p*-value: 0.000155).

Both leukocortical white matter lesion volume (adjusted *p*-value: 0.00023) and leukocortical gray matter lesion volume (adjusted *p*-value: 0.0000031) together with density influenced SDMT score in pwMS. Modularity and both white matter (adjusted *p*-value: 0.000124) and gray matter leukocortical lesion volume (adjusted *p*-value: 0.000213) showed a more pronounced relationship to SDMT in pwMS.

Additionally, we found an independent association between mean strength, leukocortical white matter lesion volume and SDMT in pwMS (adjusted *p*-value: 0.000351).

**FIGURE 4:**
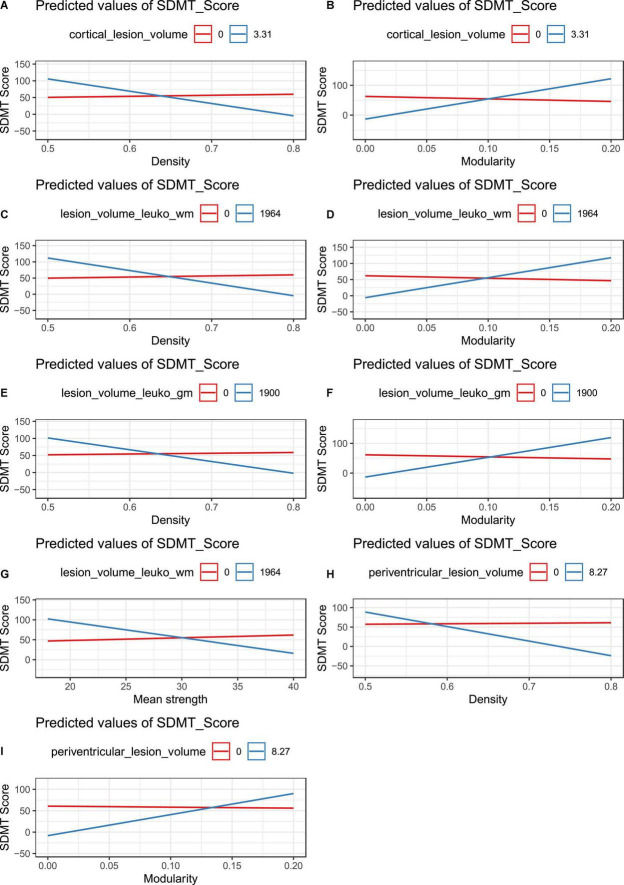
Interaction plots between symbol digit modalities test (SDMT), different lesion types, and global graph metrics in patients with multiple sclerosis (pwMS). Only significant interactions are shown. The red line shows the relationship between SDMT and network properties if no lesions are present while the blue line describes the interaction effect between high lesion volume, global graph metric, and SDMT. **(A)** Interaction between cortical lesion volume, density, and SDMT in pwMS. **(B)** Interaction between cortical lesion volume, modularity, and SDMT in pwMS. **(C)** Interaction between leukocortical white matter lesion volume, density, and SDMT in pwMS. **(D)** Interaction between leukocortical white matter lesion volume, modularity, and SDMT in pwMS. **(E)** Interaction between leukocortical gray matter lesion volume, density, and SDMT in pwMS. **(F)** Interaction between leukocortical gray matter lesion volume, modularity, and SDMT in pwMS. **(G)** Interaction between leukocortical white matter lesion volume, mean strength, and SDMT in pwMS. **(H)** Interaction between periventricular lesion volume, density, and SDMT in pwMS. **(I)** Interaction between periventricular lesion volume, modularity, and SDMT in pwMS.

Periventricular lesion volume together with density (adjusted *p*-value: 0.00003) and modularity (adjusted *p*-value: 0.000058) influenced SDMT in pwMS. For statistical details please refer to Supplementary Tables 4–12. A visualization of these interaction effects are shown in Figure 4.

### 3.8. Explorative analysis of global network metrics, lesion types and SDMT

In an explorative way we have then assessed which combination of variables best correlated with SDMT in CPMS and CIMS.

Similar as in pwMS, CPMS showed an independent association between (i) cortical lesion volume and SDMT and (ii) network density/modularity and SDMT (*p* ≤ 0.05 for all).

The volume of the white matter part of leukocortical lesions influenced the relationship between density/modularity and SDMT in CPMS patients but not in CIMS patients (*p* ≤ 0.05).

For statistical details please refer to Supplementary Tables 13–16.

## 4. Discussion

Our study shows that specific MS lesions (leukocortical and periventricular) affect the association between network metrics (density, modularity, and mean strength) and SDMT in pwMS, providing evidence that focal cortical and periventricular damage influence cognitive performance through global network properties. Nevertheless, our results also suggest that this holds true for cognitively preserved but not for cognitively impaired MS patients, despite the higher periventricular and leukocortical lesion burden of the latter group. These results extend our knowledge about the mechanisms leading to cognitive performance in patients suffering from MS, and show that deficits in processing speed are not linearly related to global network functions.

We used microstructure-weighted connectomics to assess brain network metrics in a group of pwMS with and without impairment in information processing speed, as well as in HC. We opted for this method and not for the usual count of the number of streamlines, because it is quantitative, and it has shown to be more sensitive to MS pathology than the first (Bosticardo et al., 2021).

Global connectomes were weighted using the intra-neurite volume fraction obtained with the Spherical Mean Technique (Kaden et al., 2016), which was previously related to axonal damage (Bosticardo et al., 2021). In this cohort, we found a decrease in the four global network metrics and an increase in modularity in pwMS compared to HC as it has been previously shown (Kocevar et al., 2016; Fleischer et al., 2019; Bosticardo et al., 2021) which might be plausibly the consequence of axonal degeneration processes. Interestingly, however, cognitively impaired MS patients (CIMS) did not show differences in global network metrics when compared to CPMS.

Adding to previous knowledge (Charalambous et al., 2019; Welton et al., 2020; Zhang et al., 2021), we also provide evidence that information processing speed is related to global network properties in both HC and pwMS. The correlations varied between groups (Figure 3) with the strongest associations with SDMT and clustering coefficient, efficiency, and mean strength in CIMS compared to the whole patient group and HCs. Yet, the modest nature of this relationship suggests that confounders such as the time during the day when testing was performed, psychoactive medication, stress-level, and mood might have contributed to the measured cognitive performance and hence influenced the strength of the correlations.

In pwMS, we found that a low SDMT was associated with a high modularity and a low clustering coefficient, which might appear counter intuitive. In reality, however, this is quite a plausible scenario in a disease like MS because it indicates that worse cognitive performance is associated to the segregation of the brain networks into modules (high modularity) and a low density of “triangle” pathways vs. total connected nodes [triangle and 2 ways pathways (low clustering coefficient)].

Our data cast light on the complex relationship between MS lesion type and size, global network metrics and the performance in information processing speed in pwMS.

Previous studies showed that lesion number and volume influence information processing speed in pwMS (Lazeron et al., 2000; Papadopoulou et al., 2013; Louapre et al., 2016; Forslin et al., 2018) and that there is a relationship between global network metrics and cognition (Shu et al., 2016; Welton et al., 2020), and between global network metrics and lesion number/volume in pwMS (Tozlu et al., 2020; Rocca et al., 2021). To date, however, there has been no investigation attempting at elucidating the complex relationship between focal damage, brain networks and cognition.

We found that leukocortical and periventricular lesion volume affect the relationship between global networks metrics and the performance in information processing speed in pwMS. Larger lesions may possibly impact fiber bundles to a higher degree than multiple small lesions, and in a way that is more challenging to compensate.

Especially the interaction between leukocortical lesions and global network metrics such as modularity and density were associated with SDMT outcomes. Due to their location in the lower part of the cortex and superficial white matter, leukocortical lesions affect short association fibers connecting two cortical gyri below the cortex (i.e., U-fibers) (Caucheteux et al., 2015), whose disruption has been previously associated with memory and executive function impairment (Miki et al., 1998; Rovaris et al., 2000). Our results confirm previous findings reporting an association between juxtacortical lesions and cognitive deficits in pwMS (Lazeron et al., 2000; Louapre et al., 2016; Shaaban et al., 2021), and further elucidate the mechanisms underlying this association. Furthermore, our data support previous evidence that SDMT reflects global cognition performances, encompassing not only information processing speed but also attention and working memory (Markowitsch and Tulving, 1994; Calabrese et al., 1997).

An association was also found for periventricular lesions and global network metrics on SDMT. To date, there are only limited studies about the relationship between periventricular lesions and cognition (Tiemann et al., 2009), mostly with patients suffering from dementia or other neuropsychiatric disorders (de Groot et al., 2001). Our work provides new evidence of their role in modulating the association between global network metrics and information processing speed.

Notably, intracortical lesions appeared not to have a significant impact on SDMT in line with previous findings (Nelson et al., 2011), and did not disrupt global network metrics. This may well depend on the low sensitivity of 3T MP2RAGE to this lesion type (Kober et al., 2012) and/or to the small volume of those lesions compared to leukocortical ones.

Very interestingly, an exploratory analysis showed there was no association between network metrics and SDMT performance in cognitively impaired MS patients, nor a significant interaction with lesion types. This might suggest that the pathological worsening of SDMT is not linearly related to the worsening of global network function, despite CIMS patients had larger WM and cortical lesion load than CPMS patients. These data suggest that other mechanisms (e.g., disruption of local networks, loss of compensatory processes) might be responsible for the development of processing speed deficits. Longitudinal data are needed to further investigate these processes in CIMS.

Strengths of our study include the availability of an advanced MRI protocol providing sensitivity to both cortical lesions as well as the opportunity to derive network metrics from microstructure-weighted connectomes. On the other hand, this work has also limitations including the lack of sensitivity to subpial lesions and the low representation of patients suffering from progressive MS, which did not allow us to compare among different MS phenotypes as it was performed in previous studies (Kocevar et al., 2016; Muthuraman et al., 2016). Future studies should further investigate this aspect in larger cohort.

In summary, our study provides novel insights about the mechanisms leading to alteration in information processing speed, a cognitive function that is often altered in pwMS since early stages of the disease. Network-based measures are promising prognostic biomarkers that have the potential to be used in the clinic not only to predict disease evolution, cognitive impairment and fatigue but also to guide clinicians in the early diagnostic phase and in treatment choices (Colato et al., 2021; Fleischer et al., 2021). Future work should focus on investigating the cognitive correlate of local network disruptions in pwMS.

## Data availability statement

The original contributions presented in this study are included in the article/Supplementary material, further inquiries can be directed to the corresponding author.

## Ethics statement

The Ethic Review Committee of the University of Basel approved the INsIDER study (registration number NCT05177523) and all participants signed informed consent.

## Author contributions

CG, PC, ALW, and SaS contributed to conception and design of the study. MB, SB, AD, SiS, MW, RR, P-JL, and AC performed MRI data analysis. ALW performed the neurocognitive examinations and wrote the first draft of the manuscript. SB, SaS, and ALW performed statistical analysis. All authors contributed to the manuscript revision and read and approved the submitted version.

## Footnotes

1
http://surfer.nmr.mgh.harvard.edu

2
https://github.com/ekaden/smt

3
https://sites.google.com/site/bctnet/
