# Supplementary material for: An investigation of the association between focal damage and global network properties in cognitively impaired and cognitively preserved patients with multiple sclerosis
Source: Front Neurosci. 2023 Feb 7;17:1007580. doi: 10.3389/fnins.2023.1007580 (PMC9941549; doi:10.3389/fnins.2023.1007580)
Supplement: Supplementary file 1 [file Data_Sheet_1.docx]

**SUPPLEMENT**

*Table S1.* Descriptive Analysis of Demographics and Clinical Variables in CPMS, CIMS and HC

| Variables | Healthy controls (HC) | p-values  HC vs CPMS | Cognitively Preserved MS (CPMS) | p-values  CPMS vs CIMS | | Cognitively Impaired MS  (CIMS) | p- values  HC vs CIMS |
| --- | --- | --- | --- | --- | --- | --- | --- |
| N/Gender | 92 (48 females) | p<0.05 | 50 (30 females) | p<0.05 | 18 (12 females) | | p<0.05 |
| Age | 36.2 (12.9) | p<0.05 | 42.0 (13.4) | p<0.01 | 60.3 (6.43) | | p<0.01 |
| Education (years) | 17.7 (3.63) | p=0.219 | 17 (4.98) | p=0.0928 | 14.9 (2.92) | | p<0.001 |
| EDSS |  |  | 2.25 (1.41) | p<0.001 | 4.64 (1.45) | |  |
| Disease duration in years |  |  | 8.42 (10.1) | p<0.001 | 19.2 (12.5) | |  |
| SDMT score (*) | 64.1(13.0) | p= 0.5166 | 62.8 (11.6) | p<0.001 | 38.1 (8.59) | | p<0.001 |
| SDMT z-score | -0.0978 (1.04) | p= 0.6793 | -0.02 (1.06) | p<0.001 | -1.94 (0.566) | | p<0.001 |

Abbreviations: EDSS= Expanded Disability Status Scale; SDMT= Symbol Digit Modalities Test. EDSS was measured with Expanded Disability Status Scale (27). SDMT z-Scores were calculated with standardized tables from Smith (24) controlling for age and education. For age, years of education, SDMT score, SDMT z-score and disease duration mean, and standard deviations were calculated. We have calculated p-values with t-tests if variables followed normal distribution (*) and with Mann-Whitney U- test if they did not follow normal distribution.

*Table S2.* MS phenotypes in cognitively preserved and cognitively impaired MS patients

|  | | Cognitively Preserved MS (CPMS) | | Cognitively Impaired MS (CIMS) | | | |
| --- | --- | --- | --- | --- | --- | --- | --- |
|  |  | N | % | | N | % |  |
| MS-phenotype | RRMS | 40 | 80 | | 5 | 27.7 |  |
|  | PPMS | 3 | 6 | | 7 | 38.8 |  |
|  | SPMS | 6 | 12 | | 6 | 33.3 |  |
|  | Missing | 1 | 2 | |  |  |  |
| Total |  | 50 | 100 | | 18 | 100 |  |

Abbreviations: MS: Multiple Sclerosis patients, RRMS: Relapsing-Remitting Multiple Sclerosis,

PPMS: Primary Progressive Multiple Sclerosis, SPMS: Secondary Progressive Multiple Sclerosis

*Table S3. Specific comparisons between HC, CPMS and CIMS and each global network*

| Variables | | contrasts | estimate | Standard error | t-ratio | p-value | Adjusted p-values | Multiple $\boldsymbol{R}^{\boldsymbol{2}}$  and adjusted $\boldsymbol{R}^{\boldsymbol{2}}$ | | |
| --- | --- | --- | --- | --- | --- | --- | --- | --- | --- | --- |
| Density | HC-CIMS | | 0.0364 | 0.01230 | 2.955 | 0.0052^b^ | 0.0108^b^ | | 0.1981  (0.1774) | |
|  | HC-CPMS | | 0.0196 | 0.00685 | 2.854 | 0.0066^a^ | 0.0108^b^ | |  |  |
|  | CIMS-CPMS | | -0.0168 | 0.01436 | -1.170 | 0.2438 | 0.2438 | |  |  |
| Efficiency | HC-CIMS | | 0.0234 | 0.00796 | 2.939 | 0.0027^b^ | 0.0114^a^ | | 0.5386  (0.5237) | |
|  | HC-CPMS | | 0.0108 | 0.00446 | 2.429 | 0.0163^a^ | 0.0325^a^ | |  |  |
|  | CIMS-CPMS | | -0.0126 | 0.00867 | -1.449 | 0.1495 | 0.1495 | |  |  |
| Clustering Coefficient | HC-CIMS | | 0.0222 | 0.00785 | 2.828 | 0.0053^b^ | 0.0159^a^ | | 0.5591  (0.5448) | |
|  | HC-CPMS | | 0.0101 | 0.00431 | 2.345 | 0.0203^a^ | 0.0406^a^ | |  |  |
|  | CIMS-CPMS | | -0.0121 | 0.00856 | -1.412 | 0.1599 | 0.1599 | |  |  |
| Mean strength | HC-CIMS | | 1.950 | 0.516 | 3.782 | 0.0002^b^ | 0.0007^b^ | | 0.7567  (0.7488) |  |
|  | HC-CPMS | | 0.789 | 0.306 | 2.577 | 0.0109^a^ | 0.0218^a^ | |  |  |
|  | CIMS-CPMS | | -1.161 | 0.557 | -2.082 | 0.0390^a^ | 0.0390^a^ | |  |  |
| Modularity | HC-CIMS | | 0.00116 | 0.00377 | 0.307 | 0.7590 | 0.7590 | | 0.767  (0.7594) |  |
|  | HC-CPMS | | - 0.00269 | 0.00184 | -1.463 | 0.1455 | 0.4366 | |  |  |
|  | CIMS-CPMS | | -0.00385 | 0.00406 | -0.947 | 0.3450 | 0.6901 | |  |  |

For each global graph metric, pairwise comparisons between groups were calculated. Global graph metrics differed between healthy controls and cognitively impaired MS patients and between healthy controls and cognitively preserved MS patients. Except for modularity, these comparisons were highly significant. Adjusted p-values using holm correction are reported. Abbreviations: HC: healthy controls, CPMS: cognitively preserved Multiple Sclerosis patients, CIMS: cognitively impaired Multiple Sclerosis patients. SE: standard error. ^a^ Significantly different at p $\leq$ 0.05, ^b^ Significantly different at p$\leq$0.01

Tables S4-12 display R output of significant interactions of different anatomical lesion types and global network metrics to explain cognitive performance in pwMS.

*Table S4: Interaction of cortical lesion volume and density to explain SDMT score in pwMS*

| Coefficients | Estimate | Std. Error | 95% CI | t-value | p-value |
| --- | --- | --- | --- | --- | --- |
| Intercept | 7.169e+01 | 2.856e+01 | 14.576−128.799 | 2.510 | 0.0147 * |
| Cortical lesion volume | 7.681e+01 | 3.560e+01 | 5.629−147.993 | 2.158 | 0.0349 * |
| Density | 3.077e+01 | 3.872e+01 | -46.667−108.199 | 0.794 | 0.4300 |
| Sex | -3.065e+00 | 3.205e+00 | -9.474−3.343 | -0.957 | 0.3426 |
| Education | -7.544e-03 | -3.266e-01 | -0.6605−0.645 | -0.023 | 0.9816 |
| Age | -6.709e-01 | 1.157e-01 | -0.902− -0.439 | -5.797 | 2.55e-07 *** |
| Cortical lesion volume: Density | -1.203e+02 | -5.640e+01 | -233.113− -7.564 | -2.134 | 0.0369 * |

Multiple R-squared: 0.4411, Adjusted R-squared: 0.3861, F-statistic: 8.024 on 6 and 61 DF, adjusted p-value: 0.00038178

*Table S5: Interaction of cortical lesion volume and modularity to explain SDMT score in pwMS*

| Coefficients | Estimate | Std. Error | 95% CI | t-value | p-value |
| --- | --- | --- | --- | --- | --- |
| Intercept | 99.9774 | 11.0606 | 77.860−122.094 | 9.039 | 7.34e-13 * |
| Cortical lesion volume | -23.0310 | 9.7239 | -42.475− -3.586 | -2.368 | 0.0210 * |
| Modularity | -85.0126 | 79.6554 | -244.292−74.268 | -1.067 | 0.2901 |
| Sex | -3.1118 | 3.1362 | -9.383−3.159 | -0.992 | 0.3250 |
| Education | -0.0569 | 0.3225 | -0.702−0.588 | -0.177 | 0.8605 |
| Age | -0.6657 | 0.1158 | -0.897− -0.434 | -5.746 | 3.11e-07 * |
| Cortical lesion volume: Modularity | 230.0979 | 91.0085 | 48.115−412.08 | 2.528 | 0.0141 * |

Multiple R-squared: 0.4587, Adjusted R-squared: 0.4055, F-statistic: 8.615 on 6 and 61 DF, adjusted p-value: 0.000154854

*Table S6: Interaction of white matter leukocortical lesion volume and density to explain SDMT*

*score in pwMS*

| Coefficients | Estimate | Std. Error | 95% CI | t-value | p-value |
| --- | --- | --- | --- | --- | --- |
| Intercept | 70.2414 | 27.6275 | 14.996−125.486 | 2.542 | 0.0136 * |
| Leukocortical lesion volume: WM | 0.13939 | 0.0596 | 0.020−0.258 | 2.338 | 0.0227* |
| Density | 34.2781 | 37.7090 | -41.125−109.682 | 0.909 | 0.3669 |
| Sex | -3.2704 | 3.1556 | -9.580−3.039 | -1.036 | 0.3041 |
| Education | -0.0166 | 0.3238 | -0.664−0.630 | -0.051 | 0.9593 |
| Age | -0.6884 | 0.1132 | -0.914− -0.462 | -6.081 | 8.55e-08 *** |
| Leukocortical lesion volume: WM: Density | -0.2152 | 0.0942 | -0.403− -0.026 | -2.283 | 0.0259 * |

Multiple R-squared: 0.4508, Adjusted R-squared: 0.3968, F-statistic: 8.345 on 6 and 61 DF, adjusted p-value: 0.00023328 Abbreviation: WM: white matter

*Table S7: Interaction of grey matter leukocortical lesion volume and density to explain SDMT score in pwMS*

| Coefficients | Estimate | Std. Error | 95% CI | t-value | p-value |
| --- | --- | --- | --- | --- | --- |
| Intercept | 77.2888 | 28.3061 | 13.948−131.907 | 2.730 | 0.00826 ** |
| Leukocortical lesion volume: GM | 0.1227 | 0.0626 | 0.017−0.260 | 1.958 | 0.05475 |
| Density | 22.7660 | 38.2552 | -64.671−96.183 | 0.595 | 0.55397 |
| Sex | -3.2135 | 3.2293 | -8.162−5.667 | -0.995 | 0.32362 |
| Education | -0.0126 | 0.3279 | -0.910−0.372 | 0.093 | 0.96935 |
| Age | -0.6764 | 0.1157 | -0.622− -0.077 | -5.844 | 2.13e-07*** |
| Leukocortical lesion volume: GM: Density | -0.1935 | 0.0996 | -0.412− -0.024 | -1.942 | 0.05676 |

Multiple R-squared: 0.4336, Adjusted R-squared: 0.3779, F-statistic: 7.784 on 6 and 61 DF, adjusted p-value: 0.000003083

Abbreviation: GM: gray matter

*Table S8: Interaction of white matter leukocortical lesion volume and modularity to explain SDMT Score in pwMS*

| Coefficients | Estimate | Std. Error | 95% CI | t-value | p-value |
| --- | --- | --- | --- | --- | --- |
| Intercept | 100.0648 | 10.9736 | 78.121−122.008 | 9.119 | 5.38e-13 *** |
| Leukocortical lesion volume: WM | -0.0345 | 0.0154 | -0.065− -0.003 | -2.235 | 0.0291 * |
| Modularity | -76.1541 | 75.6263 | -227.378−75.069 | -1.007 | 0.3179 |
| Sex | -3.3693 | 3.1124 | -9.593−2.854 | -1.083 | 0.2833 |
| Education | -0.0468 | 0.3205 | -0.687−0.594 | -0.146 | 0.8842 |
| Age | -0.6823 | 0.1139 | -0.910− -0.454 | -5.989 | 1.22e-07 *** |
| Leukocortical lesion volume:WM: Modularity | 0.3537 | 0.1386 | 0.076−0.630 | 2.551 | 0.0133 * |

Multiple R-squared: 0.4629, Adjusted R-squared: 0.41, F-statistic: 8.761 on 6 and 61 DF, adjusted p-value: 0.000124308

Abbreviation: WM: white matter

*Table S9: Interaction of grey matter leukocortical lesion volume and modularity to explain SDMT Score in pwMS*

| Coefficients | Estimate | Std. Error | 95% CI | t-value | p-value |
| --- | --- | --- | --- | --- | --- |
| Intercept | 98.9942 | 11.0652 | 76.867−121.120 | 8.946 | 1.05e-12 *** |
| Leukocortical lesion volume: GM | -0.0394 | 0.0174 | -0.074− -0.004 | -2.266 | 0.027 * |
| Modularity | -69.6666 | 78.6785 | -226.994−87.660 | -0.885 | 0.379 |
| Sex | -3.2827 | 3.1552 | -9.591−3.026 | -1.044 | 0.302 |
| Education | -0.03642 | 0.3234 | -0.683−0.610 | -0.113 | 0.911 |
| Age | -0.6722 | 0.1156 | -0.903− -0.440 | -5.812 | 2.41e-07 *** |
| Leukocortical lesion volume: GM: Modularity | 0.3857 | 0.1615 | 0.962−0.708 | 2.388 | 0.020 * |

Multiple R-squared: 0.4525, Adjusted R-squared: 0.3987, F-statistic: 8.404 on 6 and 61 DF, adjusted p-value: 0.00021312

Abbreviation: GM: gray matter

*Table S10: Interaction of white matter leukocortical lesion volume and mean strength to explain SDMT Score in pwMS*

| Coefficients | Estimate | Std. Error | 95% CI | t-value | p-value |
| --- | --- | --- | --- | --- | --- |
| Intercept | 72.5739 | 18.6786 | 35.223−1.099e+02 | 3.885 | 0.000254 *** |
| Leukocortical lesion volume: WM | 0.0694 | 0.0323 | 0.0048−1.34e-01 | 2.149 | 0.035611 * |
| Mean strength | 0.6412 | 0.4678 | -0.294−1.576 | 1.371 | 0.175492 |
| Sex | -3.4748 | 3.1507 | -9.775−2.825 | -1.103 | 0.274410 |
| Education | 0.0060 | 0.3230 | -0.639−6.508 | 0.019 | 0.985056 |
| Age | -0.6692 | 0.1153 | -0.899− -0.438 | -5.804 | 2.49e-07 *** |
| Leukocortical lesion volume: WM: Mean strength | -0.0023 | 0.0011 | -0.004− -3.372e-05 | -2.029 | 0.046803 * |

Multiple R-squared: 0.4427, Adjusted R-squared: 0.3879, F-statistic: 8.078 on 6 and 61 DF, adjusted p-value: 0.00035154

Abbreviation: WM: white matter

*Table S11: Interaction of periventricular lesion volume and density to explain SDMT Score in pwMS*

| Coefficients | Estimate | Std. Error | 95% CI | t-value | p-value |
| --- | --- | --- | --- | --- | --- |
| Intercept | 85.2324 | 31.7445 | 21.755−148.709 | 2.685 | 0.00933 * |
| Periventricular lesion volume | 27.3293 | 9.8576 | 7.617−47.041 | 2.772 | 0.00737 * |
| Density | 13.2050 | 46.0305 | -78.838−105.248 | 0.287 | 0.77518 |
| Sex | -3.8041 | 3.0204 | -9.843−2.235 | -1.259 | 0.21265 |
| Education | -0.0338 | 0.3160 | -0.665−0.598 | -0.107 | 0.91502 |
| Age | -0.5984 | 0.1151 | -0.828− -0.368 | -5.196 | 2.49e-06 * |
| Periventricular lesion volume: Density | -46.9971 | 15.9837 | -78.958− -15.035 | -2.940 | 0.00463 * |

Multiple R-squared: 0.4885, Adjusted R-squared: 0.4382, F-statistic: 9.711 on 6 and 61 DF, adjusted p-value: 0.000030888

*Table S12: Interaction of periventricular lesion volume and modularity to explain SDMT Score in pwMS*

| Coefficients | Estimate | Std. Error | 95% CI | t-value | p-value |
| --- | --- | --- | --- | --- | --- |
| Intercept | 97.1356 | 12.8417 | 71.456−122.814 | 7.564 | 2.48e-10 * |
| Periventricular lesion volume | -8.3265 | 2.8900 | -14.105− -2.547 | -2.881 | -0.00546 * |
| Modularity | -22.8427 | 100.5541 | -223.913−178.227 | -0.227 | 0.82105 |
| Sex | -3.9271 | 3.0619 | -10.049−2.1954 | -1.283 | 0.20449 |
| Education | -0.0802 | 0.3240 | -0.728−0.567 | -0.248 | 0.80523 |
| Age | -0.6167 | 0.1168 | -0.850− -0.283 | -5.280 | 1.82e-06 * |
| Periventricular lesion volume: Modularity | 62.2143 | 25.6339 | 10.956−113.475 | 2.427 | 0.01819 * |

Multiple R-squared: 0.4769, Adjusted R-squared: 0.4254, F-statistic: 9.267 on 6 and 61 DF, adjusted p-value: 0.000058806

Tables S13-16 display R output of significant interactions of different anatomical lesion types and global network metrics to explain cognitive performance of SDMT in CPMS

*Table S13: Interaction of cortical lesion volume and density to explain SDMT score in CPMS*

| Coefficients | Estimate | Std. Error | 95% CI | t-value | p-value |
| --- | --- | --- | --- | --- | --- |
| Intercept | 75.3875 | 29.9104 | 15.067−135.707 | 2.520 | 0.0155 * |
| Cortical lesion volume | 80.4728 | 37.7680 | 4.306−156.639 | 2.131 | 0.0389 * |
| Density | 11.6710 | 40.7870 | -70.583−93.925 | 0.286 | 0.7761 |
| Sex | -1.2690 | 3.4536 | -8.233−5.695 | -0.367 | 0.7151 |
| Education | -0.2609 | 0.3202 | -0.906−0.384 | -0.815 | 0.4197 |
| Age | -0.3387 | 0.1365 | -0.613− -0.063 | -2.481 | 0.0171 * |
| Cortical lesion volume: Density | -128.6213 | 60.5238 | -250.67− -6.5634 | -2.125 | 0.0394 * |

Multiple R-squared: 0.2367, Adjusted R-squared: 0.1302, F-statistic: 2.223 on 6 and 43 DF, p-value= 0.05899

*Table S14: Interaction of cortical lesion volume and modularity to explain SDMT score in CPMS*

| Coefficients | Estimate | Std. Error | 95% CI | t-value | p-value |
| --- | --- | --- | --- | --- | --- |
| Intercept | 87.3876 | 11.8911 | 63.404−111.368 | 7.349 | 4.03e-09 *** |
| Cortical lesion volume | -21.7223 | 11.0030 | -43.911−0.467 | -1.974 | 0.0548 |
| Modularity | -42.9976 | 84.5502 | -213.509−127.514 | -0.509 | 0.6137 |
| Sex | -1.3480 | 3.4598 | -8.325−5.629 | -0.390 | 0.6987 |
| Education | -0.2722 | 0.3219 | -0.921−0.376 | -0.846 | 0.4024 |
| Age | -0.3502 | 0.1386 | -0.629− -0.07 | -2.526 | 0.0153 * |
| Cortical lesion volume: Modularity | 202.9441 | 98.3352 | 4.632−406.256 | 2.064 | 0.0451 * |

Multiple R-squared: 0.2376, Adjusted R-squared: 0.1313, F-statistic: 2.234 on 6 and 43 DF, p-value= 0.05787

*Table S15: Interaction of white matter leukocortical lesion volume and density to explain SDMT score in CPMS*

| Coefficients | Estimate | Std. Error | 95% CI | t-value | p-value |
| --- | --- | --- | --- | --- | --- |
| Intercept | 72.9281 | 29.2455 | 13.948−131.907 | 2.494 | 0.0166* |
| Leukocortical lesion volume: WM | 0.1385 | 0.0602 | 0.017−0.2600 | 2.299 | 0.0264* |
| Density | 15.7560 | 39.8811 | -64.671−96.183 | 0.395 | 0.6947 |
| Sex | -1.2471 | 3.4289 | -8.162−5.667 | -0.364 | 0.7179 |
| Education | -0.2691 | 0.3179 | -0.910−0.372 | -0.847 | 0.4019 |
| Age | -0.3497 | 0.1350 | -0.622− -0.077 | -2.589 | 0.0131* |
| Leukocortical lesion volume: WM: Density | -0.2185 | 0.0962 | -0.412− -0.024 | -2.270 | 0.0282* |

Multiple R-squared: 0.2494 Adjusted R-squared: 0.1447, F-statistic: 2.382 on 6 and 43 DF, p-value= 0.04481

Abbreviation: WM: white matter

*Table S16: Interaction of white matter leukocortical lesion volume and modularity to explain SDMT Score in CPMS*

| Coefficients | Estimate | Std. Error | 95% CI | t-value | p-value |
| --- | --- | --- | --- | --- | --- |
| Intercept | 86.9059 | 11.6784 | 63.354−110.457 | 7.442 | 2.97e-09 *** |
| Leukocortical lesion volume: WM | -0.03277 | 0.0171 | -0.067−0.001 | -1.916 | 0.0620 * |
| Modularity | -36.0442 | 80.3695 | -198.124−126.036 | -0.448 | 0.6561 |
| Sex | -1.3752 | 3.4530 | -8.338−5.588 | -0.398 | 0.6924 |
| Education | -0.2732 | 0.3209 | -0.920−0.379 | -0.852 | 0.3992 |
| Age | -0.3515 | 0.1380 | -0.630− -0.073 | -2.546 | 0.0146 * |
| Leukocortical lesion volume: WM: Modularity | 0.3059 | 0.1447 | 0.013−0.597 | 2.113 | 0.0405 * |

Multiple R-squared: 0.2426, Adjusted R-squared: 0.137, F-statistic: 2.296 on 6 and 43 DF, p-value= 0.0519

Abbreviation: WM: white matter

**FIGURES**

*Figure S1*: Principal component analysis (PCA) of the five global graph metrics revealed high collinearity for efficiency and clustering coefficient (r= 0.992), modularity and density (r= -0.805) and efficiency and mean strength (r= 0.943).

*Figure S2:* Boxplots showing ranges in lesion number and volumes for different lesion types for both CPMS and CIMS patients. Bars in the middle represent median values. The whiskers show 95% confidence interval.
